# LPS promotes Th2 dependent sensitisation leading to anaphylaxis in a Pru p 3 mouse model

**DOI:** 10.1038/srep40449

**Published:** 2017-01-13

**Authors:** Maria J. Rodriguez, Ana Aranda, Tahia D. Fernandez, Nuria Cubells-Baeza, Maria J. Torres, Francisca Gomez, Francisca Palomares, James R. Perkins, Javier Rojo, Araceli Diaz-Perales, Cristobalina Mayorga

**Affiliations:** 1 Research Laboratory, IBIMA, Regional University Hospital of Malaga, UMA, Malaga, Spain; 2 Center for Plant Biotechnology and Genomic (UPM-INIA), Madrid, Spain; 3 Allergy Unit, IBIMA, Regional University Hospital of Malaga, UMA, Malaga, Spain; 4 Glycosystems Laboratory, Instituto de Investigaciones Químicas (IIQ), CSIC - Universidad de Sevilla, Sevilla, Spain

## Abstract

Pru p 3 is the major peach allergen in the Mediterranean area. It frequently elicits severe reactions, limiting its study in humans, raising the need for animal models to investigate the immunological mechanisms involved. However, no anaphylaxis model exists for Pru p 3. We aimed to develop a model of peach anaphylaxis by sensitising mice with Pru p 3 in combination with lipopolysaccharide (LPS) as an adjuvant. Four groups of mice were sensitised intranasally: untreated; treated with Pru p 3; treated with LPS; treated with Pru p 3 + LPS. After sensitisation mice were intraperitoneally challenged with Pru p 3 and *in vivo* and *in vitro* parameters were evaluated. Only mice in the Pru p 3 + LPS group showed anaphylaxis symptoms, including a decrease in temperature. Determination of *in vitro* parameters showed a Th2 response with an increase of Pru p 3-specific IgE and IgG1. Moreover, at the cellular level, we found increased levels of IgE and IgG1 secreting Pru p 3-specific cells and a proliferative CD4^+^ T-cell response. These results demonstrate that Pru p 3-specific anaphylaxis can be generated after nasal sensitisation to Pru p 3 in combination with LPS. This is a promising model for evaluating food allergy immunotherapies.

Food allergic reactions are an increasing worldwide problem, with important effects on health systems and the quality of life of individuals. Plant allergens are the most frequent elicitors of food allergy in adults^1^,^2^. Only a small number of protein families contain allergens^3^, one of the most important being lipid transfer proteins (LTP) from the Rosaceae family, which includes apple and peach. Allergens from this family are frequently involved in allergic reactions to plant-derived foods^4^,^5^.

The immunological mechanisms underlying food allergy are characterised by the induction of specific Th2 cells and production of specific IgE antibodies to food proteins^6^. Animal models have been used to improve our understanding of the immunological and pathophysiological mechanisms involved in the development of food allergy^6^,^7^,^8^ and to assess the modulation of the immune response^9^. The mouse immune system is well-characterised and it represents an excellent model to study an immune reaction in its full complexity *in vivo*. Various mouse models that mimic allergic responses involving specific IgE production have been developed using antigens such as ovalbumin^10^, as well as food allergens such as milk^11^, wheat (gliadin)^12^, peanut^13^ and tree nuts^14^. The allergen administration route is very important for sensitisation in food allergen models and oral administration is often seen as the logical choice^15^. However, this route may in fact have a low sensitising potential for inducing IgE antibody responses, probably due to the fact that the gastrointestinal tract has an important tolerogenic role^16^,^17^,^18^. Moreover, clinical studies have suggested that exposure to allergens through the skin or the respiratory tract might also play a role in food sensitisation^19^,^20^.

LTPs are panallergens with high stability to heat, pH and enzymatic digestion, and frequently associated with systemic and severe symptoms like anaphylaxis^21^,^22^,^23^. Pru p 3 is a member of the LTP family and the major peach allergen in the Mediterranean area^21^,^24^. The high sensitisation to Pru p 3, its cross-reactivity with other foods and pollens and its frequent involvement in severe reactions make it difficult to investigate the mechanisms underlying peach allergy in humans, although several studies have been performed looking into the role of sIgE and its influence on reaction severity^25^ and cross-reactivity with other plants^21^,^24^,^26^. Nevertheless, there is a clear need to develop approaches for its study, such as experimental models. However, although Pru p 3 sensitised mouse models with IgE production have been developed^27^, no anaphylaxis model demonstrated by *in vivo* parameters has yet been achieved. Some authors have demonstrated that the intrinsic adjuvant activity provided by associated lipids could underlie the allergenicity of some proteins^20^,^28^. Thus, the use of adjuvants may enhance allergic sensitisation in animal models. In this sense, lipopolysaccharide (LPS), a bacterial component, represents a potential candidate since it has been shown to modulate the immune response by interacting with toll-like receptor 4 (TLR4). LPS has been shown to be able to induce both Th1 and Th2 responses depending on dosage, with low levels of LPS facilitating a Th2 response and allergic reaction^29^.

In this study we aimed to develop a mouse model of LTP peach anaphylaxis using Pru p 3 as sensitiser. This was achieved through the administration of low doses of LPS as adjuvant. The allergic response after challenge with Pru p 3 was characterised based on *in vivo* (temperature and symptom scores) and *in vitro* tests (determination of Pru p 3 specific immunoglobulins by ELISA and cellular responses in splenic cells by either immunoglobulins-secreting cell quantification, T-cell proliferation and cytokine production).

## Results

### Pru p 3 together with LPS induced anaphylaxis

**Figure 1:**
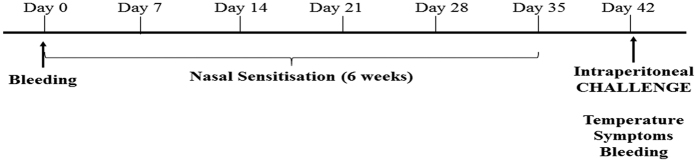
Schematic of the experimental procedures. At day 0, blood samples were collected from each mouse. Sensitisation were performed intranasally once a week for 6 weeks (on days 0, 7, 14, 21, 28, 35) with the different treatments depending on the group. At day 42 mice were challenged with one intraperitoneal dose of Pru p 3 (100 μg per mouse), following which *in vivo* parameters were measured. After this, the blood and spleen were collected from each mouse.

**Figure 2:**
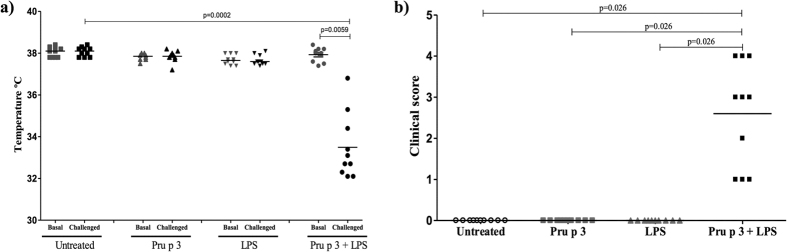
Measurement of *in vivo* parameters after challenge with Pru p 3. The appearance of systemic anaphylactic symptoms were evaluated 30–40 minutes after challenge with Pru p 3 measuring: (**a)** Changes in body temperature (°C) determining the rectal temperature before (Basal) and 30 minutes after challenge (Challenged). Statistical analysis were performed using the Wilcoxon test for related samples and significance was set at *p* < 0.05. (**b)** Clinical score, according to a scoring system: 0: no symptoms; 1: scratching and rubbing around the nose and head; 2: puffiness around the eyes and mouth, diarrhoea, ‘pilar erecti’, reduced activity and/or decreased activity with increased respiratory rate; 3: wheezing, laboured respiration, and cyanosis around the mouth and the tail; 4: no activity after prodding or tremor and convulsion and 5: death. Symbols represent individual mice in each group: White circles for untreated group (N = 10); grey squares for Pru p 3 treated group (N = 10); grey triangles for LPS treated group (N = 10) and black squares for Pru p 3 plus LPS treated group (N = 10). Statistical analysis were performed with Mann-Whitney U test for non-related samples and significance was set at *p* < 0.05.

In order to produce a mouse model of food anaphylaxis to peach, we sensitised mice intranasally with Pru p 3 (20 μg), with LPS (20 ng) or with Pru p 3 in combination with LPS (Pru p 3 + LPS) once a week for six weeks (Fig. 1). We used intranasal routes instead of oral for several reasons: (i) dose of protein needed^13^, (ii) the intrinsic tolerogenic capacity of the gastrointestinal route^16^,^17^,^30^ and (iii) the capacity of Pru p 3 to sensitise patients through inhalation^31^,^32^,^33^,^34^,^35^,^36^. One week after the final sensitisation, all mouse groups (including untreated mice) were challenged with Pru p 3 (100 μg) by intraperitoneal injection. Mice sensitised with Pru p 3 + LPS, but not those sensitised with either Pru p 3 or LPS, developed systemic anaphylaxis (Fig. 2a), consisting of a significant decrease in body temperature (p = 0.0059) and appearance of severe clinical symptoms (Fig. 2b), demonstrated by inactivity, isolation and increased respiratory rate. We did not detect any changes in the body temperature or systemic symptoms of anaphylaxis in the different control groups (untreated, sensitised with Pru p 3 or sensitised with LPS).

### Pru p 3 + LPS-exposed mice produced specific IgE and IgG1 antibodies

**Figure 3:**
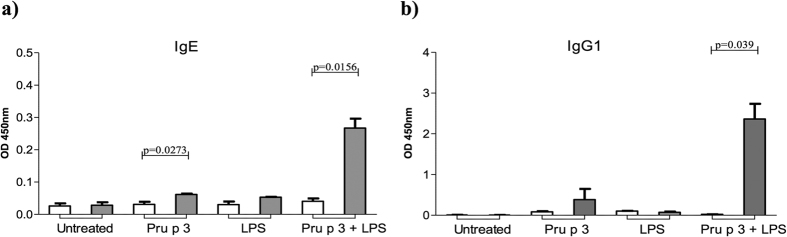
Evolution of serum level of Pru p 3-specific IgE and IgG1. Bars represent the mean level of Pru p 3-specific antibodies (**a)** IgE and (**b)** IgG1 production before (white bar) and after sensitisation (grey bar) in different groups were analysed by ELISA. Serum samples were diluted 1:8 for IgE and 1:50 for IgG1. Statistical analysis were performed using the Wilcoxon test for related samples and significance was set at *p* < 0.05.

To further explore the relationship between the *in vivo* symptoms of anaphylaxis and the humoral response, serum levels of Pru p 3-specific IgE and IgG1 were measured by ELISA (Fig. 3a,b). Pru p 3 + LPS sensitised mice developed an anaphylactic response with a significant increase in Pru p 3-specific IgE (p = 0.0156) and IgG1 (p = 0.039) production compared to basal. Data also showed a significant increase of Pru p 3-specific IgE levels (p = 0.0273) in mice sensitised with Pru p 3, although those Ievels were lower than Pru p 3 + LPS sensitised mice, which developed symptoms of an anaphylactic response. No significant changes in Pru p 3-specific IgG1 were observed in mice sensitised only with Pru p 3.

A correlation was found between anaphylactic symptoms and the change in specific IgE levels after the immunisation period, with more severe symptoms in those mice with higher levels of IgE antibodies to Pru p 3.

### Pru p 3-specific antibody secreting cells in an ELISpot assay

We assessed the number of IgE and IgG1 secreting cells after Pru p 3 stimulation by ELISpot assay. Data showed a significant increase in the number of Pru p 3-specific IgE secreting cells in mice sensitised with Pru p 3 + LPS compared to untreated, treated with Pru p 3 only, and with LPS only (p = 0.0002, p = 0.0025 and p = 0.002, respectively). Moreover, the number of Pru p 3-specific IgE secreting cells was significantly higher in mice sensitised with Pru p 3 only compared to the untreated group (p = 0.0002).

**Figure 4:**
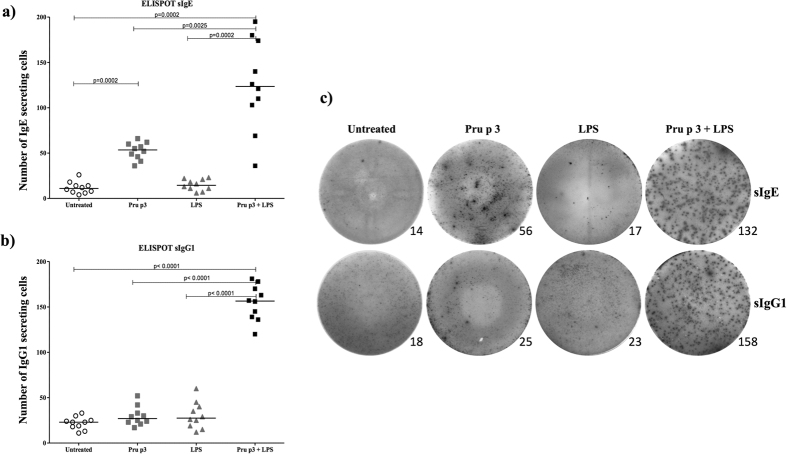
Pru p 3 specific antibody secreting cells. Specific IgE and IgG1 secreting cells were evaluated by ELISpot assay. The number of Ig secreting cells was determined by counting the formed spots using an ELISpot reader. Dots represent the number of (**a)** IgE and (**b)** IgG1 secreting splenocytes from individual mice. Symbols representing different groups of mice: White circles for untreated group (N = 10); grey squares for Pru p 3 treated group (N = 10); grey triangles for LPS treated group (N = 10) and black squares for Pru p 3 plus LPS treated group (N = 10). Statistical analysis were performed with Mann-Whitney U test for unrelated samples and significance was set at p < 0.05. Horizontal lines represent the mean values for each group. (**c)** Representative sIgE and sIgG1 ELISpot wells from Pru p 3-stimulated splenocytes from untreated mice and treated with Pru p 3, LPS and Pru p 3 + LPS. The numbers in the lower right corners indicate counted spots.

Regarding the IgG1 secreting cells, we observed a significant increase only in mice sensitised with Pru p 3 + LPS compared to untreated, treated with Pru p 3 and with LPS (p < 0.0001 for all) (Fig. 4).

### Splenocytes from Pru p 3 + LPS-exposed mice show a specific proliferative response

**Figure 5:**
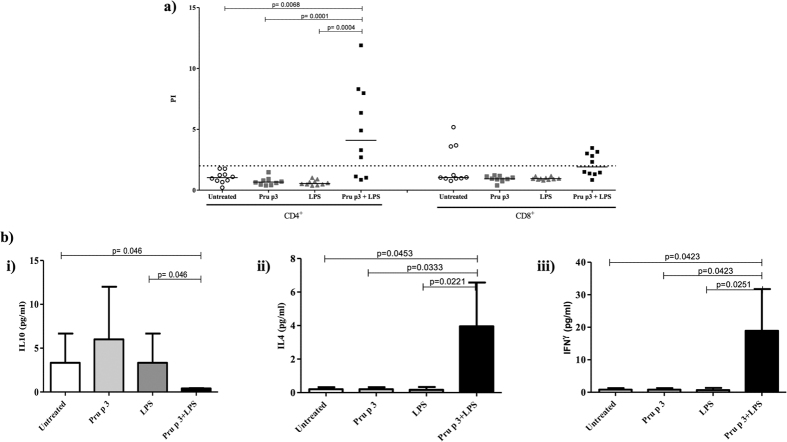
(**a**) Proliferative response of splenocytes from different groups. PI of CD4^+^ (left) and CD8^+^ subpopulations (right), after 4 days of Pru p 3 stimulation in different sensitised mice groups. PI were measured as the ratio of % CD4^+^CFSE^dim^ or CD8^+^CFSE^dim^ cells in stimulated samples/% CD4^+^CFSE^dim^ or CD8^+^CFSE^dim^ in non-stimulated samples for CD4^+^ and CD8^+^ respectively. Symbols represent individual mice in each group: White circles for untreated group (N = 10); grey squares for Pru p 3 treated group (N = 10); grey triangles for LPS treated group (N = 10) and black squares for Pru p 3 plus LPS treated group (N = 10). Statistical analyses were performed using the Mann-Whitney U test for non-related samples and significance was set at p < 0.05. Dotted line shows established cut-off point (PI = 2) for a positive test result. (**b)** Cytokine production during the proliferative response. The cytokines were quantified by ELISA in the culture supernatant obtained during the splenocyte proliferative response to Pru p 3. Bars represent the mean of concentration in pg/mL and s.d. of IL-10, IL-4 and IFN-γ secretion (i, ii and iii, respectively) in splenocytes derived from different groups of mice after re-stimulation of spleen cells with Pru p 3. White bars for the untreated group; light grey bars for the Pru p 3 treated group; dark grey bars for the LPS treated group and black bars for the Pru p 3 + LPS treated group. Statistical analysis were performed using the Mann-Whitney U test for unrelated samples and significance was set at p < 0.05.

The generation of IgE antibodies resulting in clinical food allergy could potentially be driven by Th2 cell subsets. We determined the effects of Pru p 3 + LPS on the immunological response in terms of the functional consequences on T cell priming. Splenocytes of mice from the different groups were isolated following challenge. We then analysed the proliferative response of CD4^+^ and CD8^+^ T cell subpopulations following 4 days of incubation with Pru p 3. A significant increase in the proliferative index (PI) was only detected in the CD4^+^ T cell subpopulation, and only in the Pru p 3 + LPS group compared to untreated (p = 0.0068), Pru p 3 (p = 0.0001) and LPS (p = 0.0004) groups. It is important to note that, despite the presence of LPS, no proliferative response of CD8^+^ T cells was found. These results indicate that, although LPS is usually related to a Th1 response in which the CD8^+^ T cell subpopulation is involved, the sensitisation of mice with low LPS doses in the presence of Pru p 3 leads to a Th2 response (Fig. 5a).

In order to characterise the proliferative response at the molecular level, we evaluated the production of different cytokines: IL-10, indicative of the regulatory response; IL-4, indicative of the Th2 response; and IFN-γ indicative of the Th1 response. These cytokines were quantified in the culture supernatant obtained during the splenocyte proliferative response to Pru p 3 by ELISA (Fig. 5b). Data showed a significant increase in production of IL-4 and IFN-γ in mice immunised with Pru p 3 + LPS compared to untreated, treated with Pru p 3 only, and LPS only (IL-4: p = 0.0453, p = 0.0333 and p = 0.0221 respectively; IFN-γ: p = 0.0423, p = 0.0423 and p = 0.0251, respectively). Moreover, the group of mice immunised with Pru p 3 + LPS was the only one with no IL10 production (Fig. 5b). Interestingly, an IL-4-driven increase in splenocytes of the group sensitised only with Pru p 3 was not observed, suggesting that the mechanism involved in the induction of Th2 responses is dependent on Pru p 3 in the presence of LPS.

## Discussion

The development of Th2 cells is believed to be a critical step in the underlying mechanisms of food allergy. However, the factors involved in the triggering of food allergen-specific Th2 activation remain poorly understood. Pru p 3 is one of the most important plant food panallergens in the Mediterranean area and can cause life-threatening reactions^21^,^24^,^26^. The severity of the reactions limits the use of human studies and raises the need for animal models to assess the immunological mechanisms. However, although mouse models with Pru p 3 IgE production have been generated^27^, no comprehensive anaphylaxis model, confirmed by both *in vivo* and *in vitro* parameters, exists. The development of such a model is critical for many applications, for example to test the safety and potential benefits of immunotherapy for food allergens.

The choice of administration route is critical for the generation of an allergy model. In the case of peach allergy, although it has been associated with the oral and gastrointestinal routes, some authors found a limited IgE antibody response that could be the consequence of the tolerance mechanisms associated with these routes^16^,^17^. Moreover, food allergic reactions may also develop following airborne sensitisation and/or challenge, as shown by Pru p 3 which can sensitise patients through both the oral and inhalational routes^31^,^32^,^33^,^34^,^35^,^36^.

Some data indicated that the tendency of some proteins to trigger the adaptive immune responses towards a Th2 pattern is due to their ability to promote TLR4 signalling^28^. Moreover, the allergenicity of some proteins for inducing a Th2 response could be enhanced in the presence of adjuvants^37^,^38^. In fact, it is thought that the exposure of the mucosal immune system to bacterial compounds like LPS alongside food antigens can lead to food antigen sensitisation and allergy. These bacterial products might promote allergic sensitisation via TLR4 signalling^39^,^40^. Previous studies have shown that depending on the concentration, LPS can modulate the response towards a Th1 pattern at high concentrations or enhance the induction of a Th2 response when using low levels (<100 ng)^37^,^41^.

In the present study, we have demonstrated that the Pru p 3-specific anaphylactic response can be generated after nasal sensitisation to Pru p 3 in combination with LPS as adjuvant. This anaphylactic response was demonstrated *in vivo* by a significant temperature drop alongside the appearance of objective symptoms of anaphylaxis in the group of Pru p 3 + LPS immunised mice. This anaphylactic model leads to higher levels of humoral Th2-mediated immunoglobulins IgE and IgG1 which agree with results observed in sensitisation models for other allergens^27^, obtained using a different administration route (intraperitoneally) and adjuvant (aluminium hydroxide).

It is interesting to note that although an increase of Pru p 3-specific IgE was observed in both Pru p 3 and Pru p 3 + LPS immunised mice, a significantly higher level was observed in the latter, suggesting a key role of sIgE in the clinical response. This finding potentially indicates that sIgE levels can be a biomarker for differentiating sensitisation from real allergy as has been proposed by other authors^42^,^43^. Moreover the anaphylactic group, treated with Pru p 3 + LPS, also showed a significant increase of Pru p 3 specific IgG1 that was not observed in mice sensitised with Pru p 3 only. It is tempting to speculate that high levels of sIgE together with the increase of sIgG1 may be related with the appearance of anaphylactic symptoms as has been demonstrated in patients with anaphylaxis to peach and other allergens^25^,^44^,^45^.

The use of some adjuvants may compromise the specific response to the allergen, and moreover, the type of response. LPS has a role in the innate immune system in the development of a Th1 response^46^ in which CD8^+^ T cells are involved. However, in our model the use of LPS in combination with Pru p 3 not only induces the specific antibody response to Pru p 3 but also leads to a Th2 response pattern, confirmed by the increase of specific IgE and IgG1 antibodies, of IgE and IgG1 secreting cells, and increased IL4 levels. Moreover, the analysis of the proliferating cell phenotype showed that the combination of Pru p 3 + LPS induced Pru p 3-specific activation of CD4^+^ T-lymphocytes and no proliferative response of CD8^+^ T cells was found. Additionally, no proliferation to Pru p 3 was found in mice sensitised with LPS alone.

Our results suggest that Pru p 3 challenge of Pru p 3 + LPS mice sensitised by the nasal route can lead to anaphylactic responses. The mechanism by which Pru p 3 + LPS exposure can elicit an adaptive response, leading to the induction of IgE and CD4^+^ T cells is not clear but may relate to features of the resident dendritic cells within each site, such as differential expression of innate receptors. Additionally, previous reports have shown that Th2-type responses induced in the gastrointestinal tract can influence immunophysiological responses in distant, non-inflamed mucosal tissue and regulate airway responsiveness^47^,^48^. The priming of naïve T cells to become protein specific Th2 cells requires the activation of the innate immune system to be provoked by LPS^29^,^49^ causing IgE synthesis. The proliferative response of CD4^+^ T cells in the presence of Pru p 3 demonstrates that the results are mediated by the adaptive immune response with T cells specific to the allergen and not the early innate immune response to LPS.

Our results suggest that LPS alongside Pru p 3 can stimulate immune cells in the upper respiratory tract and trigger a state of systemic pro-inflammation. To our knowledge, this is the first effective model of anaphylaxis caused by Pru p 3. The use of these anaphylactic mice shows great potential for testing and developing novel immunotherapy approaches for food allergens, with potential applications to other allergies.

## Methods

### Mice sensitisation

Female Balb/c mice (4–5 weeks old), as used in other studies^50^,^51^, were used (Janvier Lab, Saint-Berthevin Cedex, France).

All experimental animal procedures conformed to international standards of animal welfare and were approved by the Animal Experimentation Ethics Committee of BIONAND, Malaga, Spain. Mice were divided into 4 groups (N = 10 per group): (1) Untreated; (2) Treated with Pru p 3; (3) Treated with LPS; (4) Treated with both Pru p 3 and LPS (Pru p 3 + LPS). Mice were anaesthetised with inhaled Forane (Abbott Laboratories Ltd., Kent, UK) and sensitised with six nasal administrations (12 μl) of 20 μg of purified peach protein, natural Pru p 3 (Bial Laboratory, Zamudio, Spain) and/or 20 ng of LPS (InvivoGen, San Diego, CA), depending on the group, according to published studies^38^, on days 0, 7, 14, 21, 28 and 35 (Fig. 1). Mice were challenged 1 week after the last immunisation (day 42) by receiving one intraperitoneal dose of Pru p 3 (100 μg per mouse) (Fig. 1).

### Assessment of anaphylactic responses

The appearance of systemic anaphylaxis was evaluated 30–40 minutes after challenge (day 42) in their habitual living environment that is their own cage, by measuring drop in body temperature with a rectal probe, and by assessing physical and behavioural symptoms.

Symptoms were established according to a scoring system^13^, 0: no symptoms; 1: scratching and rubbing around the nose and head; 2: puffiness around the eyes and mouth, diarrhoea, ‘pilar erecti’, reduced activity and/or decreased activity with increased respiratory rate; 3: wheezing, laboured respiration, and cyanosis around the mouth and the tail; 4: no activity after prodding or tremor and convulsion and 5: death. Mice were bled from the retro-orbital plexus on day 0 (Basal) and 42 (after challenge), and sera were stored at −20 °C for humoral studies. After the challenge and *in vivo* evaluation, mice were sacrificed by cervical dislocation and the spleen was removed aseptically and teased to prepare a single-cell suspension for cellular analysis.

### Humoral response

Pru p 3-specific serum antibody (IgE and IgG1) was measured at 0 and 42 days in mice sera by ELISA. Briefly, 96-well microtiter polystyrene ELISA plates (Corning, NY, USA) were coated with 5 μg/ml of Pru p 3 in coating buffer, pH 9.6, overnight at 4 °C. Fifty μl of serum was added in duplicates (diluted 1:8 for IgE and 1:50 for IgG1) and incubated overnight at 4 °C. After washing, the plates were incubated with biotinylated labelled goat anti-mouse IgE at 1:1000 (BD Pharmingen, San Diego, CA, USA) and goat anti-mouse IgG1 at a 1:3000 dilution (BD Pharmingen) for 1 hour at room temperature. After washing, avidin-HRP (BD Pharmingen) solution at 1:5000 for IgE and 1:1000 for IgG1 was added and samples were incubated for 30 minutes at room temperature. Then, 50 μl of ready to use TMB substrate (BD Pharmingen) was added and incubated for 10 minutes in dark condition. The enzymatic reaction was stopped with 50 μl of H_2_SO_4_(2 N) and absorbance was read at 450 nm. The ELISA results were expressed in optical density.

### ELISpot Assay

The number of antibody-secreting cells was measured by a modified ELISpot assay originally described by Czerkinsky *et al*.^52^. Briefly, ELISpot Multiscreen HTS plates (96 wells) (Millipore, Darmstadt, Germany) were coated overnight at 4 °C with Pru p 3 at 5 μg/mL. Plates were blocked for one hour at 37 °C with blocking solution (Sigma-Aldrich, St. Louis, MO) and a total of 1.5 × 10^5^ cells were seeded and incubated for 48 hours. After washing, 50 μl of alkaline phosphatase labelled- anti IgE at 1:1000, anti-IgG1 at 1:4000 and anti-IgM (positive control) at 1:3000 (SouthernBiotech) were added to each well and incubated overnight at 4 °C. After washing, 100 μl of ready to use solution of previously filtered NBT-BCIP (Sigma-Aldrich) was added. The colorimetric reaction was allowed to proceed until spots were clearly evident on positive-control, total-IgM-capture wells (15 minutes). The reagent and plate backing material were removed, and reactions were quenched with distilled water. The number of Ig secreting cells was determined by counting the formed spots using an ELISpot Bioreader(R) 6000 (BioSys, Karben, Germany).

### Lymphocyte proliferation

Proliferation analysis was performed by flow cytometry. Spleen cells were harvested and labelled with 5,6-carboxyfluorescein diacetate N-succinimidyl ester (CFSE) using CellTrace™ CFSE Cell Proliferation Kit (Life Technologies, Invitrogen, USA) following the manufacturer’s instructions. After this, 2 × 10^6^ cells/well were seeded in 24-well plates (Nunc, Roskilde, Denmark) in complete medium (RPMI 1640 (Lonza, BioWhittaker, Verviers, Belgium) supplemented with 2 mM of L-Glutamine (BioWhittaker, Pittsburgh, PA), gentamicin (5 μg/uL) (Normon, Madrid, Spain), streptomycin (50 ng/mL) and 10% fetal bovine serum (BioWhittaker)). Cells were incubated in absence (negative control) or presence of Pru p 3 at 5 μg/mL for 4 days at 37 °C and 5% of CO_2_.

After this, the cells were harvested and stained with specific fluorochrome-conjugated moAbs, antiCD4-PE and antiCD8-PE-Cy7A (BD Pharmingen) and phenotyped with a BD™ FACSCanto II flow cytometer and analysed using FlowJo^®^ software (Tree Star, Inc. USA). Results were expressed as proliferation index (PI) for CD4^+^ or CD8^+^ measured as the ratio: %CD4^+^CFSE^dim^ or %CD8^+^CFSE^dim^ in stimulated sample/%CD4^+^CFSE^dim^ or %CD8^+^CFSE^dim^ in non-stimulated sample. Proliferative response was considered positive when the PI was higher than 2.

### Cytokine production

IL-10, IL-4 and IFN-γ were determined from 72 h spleen cell culture supernatants described above using sandwich ELISA kits (BD Biosciences Pharmingen) according to the manufacturer’s instructions. The results were expressed as pg/ml.

### Statistical analysis

Data were presented as individual values and mean with s.d. values. Quantitative unrelated variables were analysed using the Mann-Whitney U test, Kruskal-Wallis test and X^2^-test. Comparisons of related samples were carried out by Wilcoxon test. Correlation analysis was performed using Pearson’s test. P-values lower than 0.05 were considered statistically significant.

## Additional Information

**How to cite this article**: Rodriguez, M. J. *et al*. LPS promotes Th2 dependent sensitisation leading to anaphylaxis in a Pru p 3 mouse model. *Sci. Rep.* **7**, 40449; doi: 10.1038/srep40449 (2017).

**Publisher's note:** Springer Nature remains neutral with regard to jurisdictional claims in published maps and institutional affiliations.
